# Controlling surface waves with temporal discontinuities of metasurfaces

**DOI:** 10.1515/nanoph-2022-0685

**Published:** 2023-01-24

**Authors:** Xuchen Wang, Mohammad S. Mirmoosa, Sergei A. Tretyakov

**Affiliations:** Institute of Nanotechnology, Karlsruhe Institute of Technology, Karlsruhe, Germany; Department of Electronics and Nanoengineering, Aalto University, Espoo, Finland

**Keywords:** amplification, frozen wave, metasurfaces, reactive boundary, surface wave, temporal discontinuity

## Abstract

Static reactive metasurfaces allow excitation and propagation of surface waves. In this paper, we theoretically elucidate how surface-wave propagation along a reactive boundary is affected by temporal discontinuities of effective parameters characterizing the boundary. First, we show that by switching the value of the surface reactance, the velocity of surface waves is fully controlled, and the power of reflected and transmitted surface waves can be amplified. Second, we indicate that when a boundary supporting waves with transverse-electric polarization is switched to the one allowing only transverse-magnetic polarization, the propagating surface wave is “frozen” and converted to a static magnetic-field distribution. Moreover, efficiently, these fields can be “melted”, restoring propagating surface waves when the boundary is switched back to the initial state. Finally, we demonstrate that temporal jumps of the boundary reactance couple free-space propagating waves to the surface wave, in an analogy to a spatial prism. All these intriguing phenomena enabled by temporal discontinuities of effective properties of reactive metasurfaces open up interesting possibilities for the generation and control of surface waves.

## 1 Introduction

Utilizing temporal discontinuities for controlling interactions of electromagnetic waves with media has recently received a remarkable attention [1]. Temporal discontinuity (or temporal boundary) means that the effective parameters of a medium are spatially uniform while they undergo a sudden change in time, which is in contrast to the spatial interface where the medium properties are discontinuous in space but uniform in time [2]. Similarly to spatial boundaries, a temporal boundary also results in the creation of reflected (backward) and transmitted (forward) waves [3], but the scattering processes conserve momentum rather than energy. Due to the conservation of momentum, the angular frequency of wave should be converted at time discontinuities of material parameters. This effect was studied theoretically [4, 5] and confirmed experimentally at microwaves [6] as well as terahertz [7] and optical frequencies [8, 9]. However, frequency conversion is not the only interesting possibility. Extending the study to more complex media (such as temporal slabs [10], anisotropic media [11–14], chiral media [15], magnetoplasma [16], etc.) rather than simple magnetoelectric ones [2], other intriguing wave phenomena have been uncovered. For example, anti-reflection temporal coatings [10, 17], temporal beam splitting [18], inverse prism [11], temporal aiming [12], polarization conversion [13], polarization splitting [15], and nonreciprocity [16] are some of these findings.

From the initial steps till now, the research focus has been mainly on unbounded bulk media. There are also several studies reporting effects at temporal discontinuities of other structures such as cavities [19, 20], leaky waveguides [21] and graphene sheets [22–24]. Meanwhile, to the best of our knowledge, temporal discontinuity of artificial two-dimensional (2D) materials, i.e., metasurfaces, has not been considered and investigated thoroughly. Metasurfaces or 2D metamaterials have already shown strong potential due to the versatility and a multitude of functionalities that they provide [25]. Temporal discontinuity as an additional degree of freedom for manipulating waves increases this capability and makes metasurfaces evolve to the next level. In this work, we focus on this subject and investigate surface waves propagating on metasurface boundaries when the effective surface impedance changes rapidly from one value to another in time (the electromagnetic properties of metasurfaces are modeled by surface impedances, susceptibilities, and polarizabilities [26]). We show that due to such temporal discontinuities, backward and forward surface waves are created, and, therefore, we derive the corresponding reflection and transmission coefficients associated with those waves. Also, we find that the phase velocity of the surface wave can be freely controlled by switching the surface reactance, in particular, realizing ultra-slow surface waves. Furthermore, we indicate that the power carried by surface waves can be amplified after the temporal jump. Moreover, interestingly, we see that if a reactive metasurface boundary is switched from a capacitive to an inductive one, the propagating surface wave is converted to a static magnetic field distribution over the boundary, and the magnetic energy is frozen. Astonishingly, by switching back to a capacitive boundary, the electric energy is restored, and the surface wave starts to propagate again. Finally, we demonstrate that a temporally discontinuous surface is able to couple a free-space propagating wave to a surface wave, acting as a temporal prism.

## 2 Dispersion diagram of time-invariant reactive boundaries

**Figure 1:**
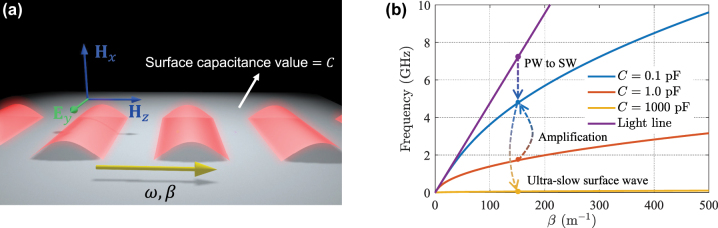
Dispersion relations of static capacitive metasurfaces. (a) A TE-polarized surface wave that is propagating along an impenetrable capacitive boundary. (b) Dispersion relations of a boundary with different surface capacitances. The dashed arrows represent the wave phenomena induced by temporal discontinuity of metasurface, which will be discussed in detail in Sections 4.1, 4.2, and 4.4. PW and SW are abbreviations of plane wave and surface wave, respectively.

Let us consider an impenetrable metasurface which is characterized by a capacitive surface impedance with a constant surface capacitance value *C*. First, we remind the well-known dispersion relation for waves traveling along such impedance boundaries in the stationary case. Since the surface impedance is capacitive, transverse electric (TE)-polarized waves are allowed to propagate along the surface, as shown in Figure 1(a). We write the electric field as **E**_*y*_ = *E*_0_e^−j*βz*−*αx*^e^j*ωt*^ **y**, where *β* is the propagation constant along the surface, *α* is the decay factor along the *x*-direction, and *E*_0_ represents the wave amplitude. The field corresponding to the surface wave exists in free space. Hence, it must satisfy the Helmholtz equation written for free space, i.e., ∇^2^ **E**_*y*_ − *μ*_0_ *ϵ*_0_∂^2^ **E**_*y*_/∂*t* ^2^ = 0. By substituting the electric field in the Helmholtz equation, we arrive at the relation *α* ^2^ − *β* ^2^ + *ω* ^2^ *ϵ*_0_ *μ*_0_ = 0. In addition, we also substitute the electric field in Faraday’s law ∇ × **E** = −*μ*_0_∂**H**/∂*t*, and, as a result, the tangential component of the magnetic field associated with the surface wave can be calculated: $H_{z}=\frac{\alpha E_{0}}{j\omega \mu_{0}}{\text{e}}^{-\text{j}\beta z-\alpha x}{\text{e}}^{\text{j}\omega t}z$ (note that TE-polarized waves have two components of magnetic field). Now, the tangential components of the fields at the boundary *x* = 0 respect the boundary condition which is j*ωC* ⋅ **E**_*y*_ = ***n*** × **H**_*z*_ (***n*** is the surface normal). Imposing this condition gives us the dispersion relation

(1) $$\beta^{2}=k_{0}^{2}\left(1+\eta_{0}^{2}\omega^{2}C^{2}\right),$$

in which $k_{0}=\omega \sqrt{\epsilon_{0}\mu_{0}}$ and $\eta_{0}=\sqrt{\mu_{0}/\epsilon_{0}}$ are the free-space wavenumber and intrinsic impedance, respectively. Figure 1(b) presents the dispersion curves for different boundaries characterized by different values of surface capacitances. As the figure explicitly shows, if the value of *C* decreases, the curve shifts towards the light line, and if the value of *C* grows, the curve shifts towards the horizontal axis (the propagation constant axis). In the following, we explain how we can efficiently use this property together with temporal discontinuities in order to engineer the phase and energy velocities of surface waves.

## 3 Forward and backward surface waves

Assume that at some moment of time (*t* = 0) the surface capacitance quickly changes from one value to another one. This temporal jump generates forward and backward surface waves. This is in analogy with temporal jumps of volumetric material parameters such as permittivity and permeability [3]. In this latter case, we know that since the material is uniform in space, the phase constant (or the propagation constant) is conserved meaning that it does not change at the moment of temporal discontinuities of material parameters, however, the angular frequency changes. Interestingly, the equation for the new angular frequency has two solutions whose absolute values are the same but they are different in sign. The positive value corresponds to a forward wave, and the negative value is related to a backward wave (there is an analogy with time reversal). Similarly, for our specific scenario, at a time jump of surface reactance, the propagation constant *β* along the surface is fixed, because the surface is assumed to be spatially uniform at all times. Thus, after the jump, we have two surface waves propagating over the surface in opposite directions. As the next step, we will find the amplitudes of these two waves.

We suppose that before the moment of capacitance jump, a TE surface wave (propagation constant *β*, frequency *ω*_0_) is propagating over a capacitive boundary, with electric and magnetic fields

(2) $$\begin{array}{cc} & E_{i}=E_{0}{\text{e}}^{-\text{j}\beta z-\alpha_{0}x}{\text{e}}^{\text{j}\omega_{0}t}y,\\ & H_{i}=-\frac{E_{0}}{\omega_{0}\mu_{0}}\left(\text{j}\alpha_{0}z+\beta x\right){\text{e}}^{-\text{j}\beta z-\alpha_{0}x}{\text{e}}^{\text{j}\omega_{0}t}. \end{array}$$

We call this wave *incident*. At *t* = 0, the surface capacitance rapidly changes from *C*_0_ to *C*_1_. In order to adapt to the new boundary, the wavenumber should be conserved and the wave angular frequency is converted to *ω*_1_, according to the dispersion relation. Thus, we have (after a short transient period)

(3) $$\begin{array}{cc} & E_{r}=R_{E}E_{0}{\text{e}}^{-\text{j}\beta z-\alpha_{1}x}{\text{e}}^{-\text{j}\omega_{1}t}y,\\ & E_{t}=T_{E}E_{0}{\text{e}}^{-\text{j}\beta z-\alpha_{1}x}{\text{e}}^{\text{j}\omega_{1}t}y, \end{array}$$

where *R*_E_ and *T*_E_ define the amplitudes of the backward and forward waves, respectively. Technically, we call them *reflection and transmission coefficients* associated with electric field. In this work, we only consider lossless surfaces. If the surface after the jump is lossy, the reflected and transmitted surface modes will propagate and also exponentially decay along the surface. Additional investigations are needed for accurate analysis of the effects of losses. Note that in the above equation, conservation of the propagation constant and change of the angular frequency result in a different value for the attenuation constant of the fields above the boundary (parameter *α*). Having the expressions of the electric field, the magnetic fields of the reflected and transmitted fields are obtained by employing Maxwell’s equations, leading to

(4) $$\begin{array}{cc} & H_{r}=\frac{R_{E}E_{0}}{\omega_{1}\mu_{0}}\left(\text{j}\alpha_{1}z+\beta x\right){\text{e}}^{-\text{j}\beta z-\alpha_{1}x}{\text{e}}^{-\text{j }\omega_{1}t},\\ & H_{t}=-\frac{T_{E}E_{0}}{\omega_{1}\mu_{0}}\left(\text{j}\alpha_{1}z+\beta x\right){\text{e}}^{-\text{j}\beta z-\alpha_{1}x}{\text{e}}^{\text{j }\omega_{1}t}. \end{array}$$

In order to derive the coefficients *R*_E_ and *T*_E_, electromagnetic boundary conditions are needed. Based on Maxwell’s equations, for free space, we have time derivatives of electric and magnetic flux densities which are equal to the curl of magnetic and electric fields, respectively. These vectors (**D** and **B**) should be continuous at the moments of abrupt changes of the surface impedance. Therefore,

(5) $$D_{t=0^{-}}=D_{t=0^{+}},\;B_{t=0^{-}}=B_{t=0^{+}}.$$

However, since the environment allowing surface-wave propagation is fixed (free space), the continuity described by the above equation results in the continuity of the electric and magnetic fields. Let us choose one point infinitesimally close to the boundary, at *x* = *δ*, where *δ* → 0^+^. First, regarding the electric field continuity, $E_{i}{|}_{t=0^{-}}=\left(E_{r}+E_{t}\right){|}_{t=0^{+}}$ , it is easy to show that *T*_E_ + *R*_E_ = 1. Second, according to the continuity of the magnetic field at the chosen point, $H_{i}{|}_{t=0^{-}}=\left(H_{r}+H_{t}\right){|}_{t=0^{+}}$ , we can also show that *T*_E_ − *R*_E_ = *ω*_1_/*ω*_0_. In fact, this identity is achieved by considering only the *x* component of the magnetic field. We do not use the *z* component of the magnetic field because it is derived based on the derivative of the electric field of the surface wave with respect to *x*. But, in this direction, there is a discontinuity in space. However, in contrast, **H**_*x*_ is obtained from the derivative of the electric field with respect to *z*, and, in this direction, there is no spatial discontinuity. Therefore, only **H**_*x*_ is required to be continuous in our calculation. Finally, since we have two unknowns and two equations, the reflection and transmission coefficients related to the electric field can be found as

(6) $$T_{E}=\frac{1}{2}\left(1+\frac{\omega_{1}}{\omega_{0}}\right),\;R_{E}=\frac{1}{2}\left(1-\frac{\omega_{1}}{\omega_{0}}\right).$$

Notice that based on Eqs. (3) and (4), we can also define reflection and transmission coefficients in terms of the *x*-component of the magnetic field, i.e., **H**_r*x*_ = *R*_H_ **H**_i*x*_ and **H**_t*x*_ = *T*_H_ **H**_i*x*_. According to this definition, we obtain that

(7) $$T_{H}=\frac{1}{2}\left(1+\frac{\omega_{0}}{\omega_{1}}\right),\;R_{H}=\frac{1}{2}\left(1-\frac{\omega_{0}}{\omega_{1}}\right).$$

It is important to note that during the short time interval when the surface parameters quickly change in time, the energy of the surface wave is partially converted also to waves propagating in space, as during the transition time the surface wave frequency is converted to various frequencies, which correspond also to propagating waves. However, as soon as the switching process is over, this coupling process stops, and in the established regime after the time discontinuity, only the eigenmode of the surface with the changed parameters survives in the vicinity of the boundary. Eqs. (6) and (7) are accurate only when the transient time is short and radiation to free space is weak compared to the eigensurface modes. Rigorous analysis of all the scattering modes (including both surface modes and free-space modes) during and after jumps needs field integration over all frequencies [21, 27], [28], [29], or the use of Laplace transform of fields [22, 30].

## 4 Various phenomena at temporal jumps of boundary properties

Next, we provide four examples of surface wave phenomena including surface wave amplification, deceleration, freezing, and surface wave excitation by plane waves. All these important phenomena confirm that temporal discontinuity is indeed an efficient tool for manipulating waves in the desired way.

### 4.1 Surface wave amplification

At a temporally varying interface, the passivity condition does not necessarily hold since energy may enter or leave the system through the external device that changes the surface properties. Here, we examine the gain of the system that is defined as the ratio of the incident and scattered powers (the power flux density integrated from *x* = 0 to *x* = +∞), i.e., *G* = (|*P*_r_| + |*P*_t_|)/|*P*_i_|. Note that the unit of power here is W/m. By substituting the electric and magnetic field expressions (Eqs. (2)–(4)), the gain parameter can be evaluated as

(8) $$\begin{array}{cc} G & =\frac{\overset{+\infty }{\underset{0}{\int }}|R\left(E_{r}\times H_{r}^{*}\right)|dx+\overset{+\infty }{\underset{0}{\int }}|R\left(E_{t}\times H_{t}^{*}\right)|dx}{\overset{+\infty }{\underset{0}{\int }}|R\left(E_{i}\times H_{i}^{*}\right)|dx}\\ & =\frac{\alpha_{0}}{2\alpha_{1}}\left(\frac{\omega_{0}}{\omega_{1}}+\frac{\omega_{1}}{\omega_{0}}\right). \end{array}$$

Clearly, gain is always larger than unity if *α*_0_ > *α*_1_, which is equivalent to *ω*_1_ > *ω*_0_ or *C*_0_ > *C*_1_. The wider is the jump, the more energy will be injected into the system. If the capacitance does not change (*ω*_0_ = *ω*_1_ and *α*_0_ = *α*_1_) at *t* = 0, then, obviously, *G* = 1. Note that for *C*_0_ < *C*_1_, according to Eq. (8), it is also possible to realize gain, but not always.

**Figure 2:**
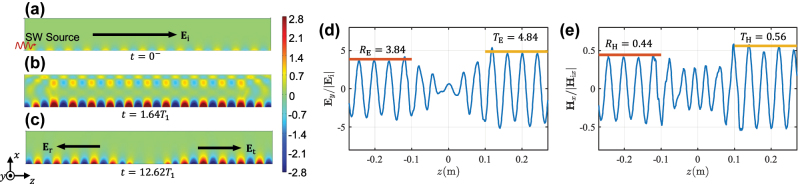
Normalized electric fields distribution (*y*-component) at (a) *t* = 0^−^, (b) *t* = 1.64*T*_1_, and (c) *t* = 12.62*T*_1_. Normalized (d) electric and (e) magnetic fields variations along the surface at *t* = 12.62*T*_1_. The red and orange lines show the theoretical values of transmission and reflection coefficients.

Next, we numerically verify the reflection, transmission, and amplification phenomena. As an example, we assume that before the jump *C*_0_ = 10 pF and after the jump *C*_1_ = 0.1 pF. The eigenfrequencies at these two states are calculated as *ω*_0_/(2*π*) = 0.556 GHz and *ω*_1_/(2*π*) = 4.83 GHz, with the same propagation constant along the surface *β* = 153 m^−1^. Figure 2(a)–(c) shows the fields simulated by employing COMSOL Multiphysics. The surface wave is launched from the left boundary of the computation domain, and it propagates to the right boundary assigned with perfect matched layer (PML). At *t* = 0^−^, the surface wave is uniformly distributed over the boundary with the surface capacitance *C*_0_ [see Figure 2(a)]. At *t* = 0 the excitation source is removed, and the boundary capacitance is switched to *C*_1_. While the new surface eigenmode is established at the changed boundary, transient scattering is generated in the form of free-space propagating waves, as seen in Figure 2(b). The transmitted and reflected surface waves travel in the opposite directions and form a standing wave. At *t* = 12.62*T*_1_ (*T*_1_ = 2*π*/*ω*_1_), the two oppositely traveling surface waves are fully separated. One can explicitly see in Figure 2(c) that the electric fields of the transmitted and reflected surface waves are much stronger than the initial incident wave in Figure 2(a). The calculated electric fields at *t* = 12.62*T*_1_ on the boundary are shown in Figure 2(d) confirming a good agreement with the theoretically predicted values. The magnetic fields (the *x*-component) of the two waves reduce to around half of the incident wave amplitude, as shown in Figure 2(e). By summing up the powers of two reversely propagating surface waves, the gain of the system can be confirmed as *G* = 4.32.

### 4.2 Ultra slow surface waves

Another possibility is to fully control the velocity of waves propagating above the boundary and to realize ultra-slow surface waves. This possibility is noteworthy when we do not have access to the excitation source for being adjusted, or it is difficult to efficiently generate slow waves directly by external sources. Let us consider the scenario of switching surface capacitance from a low to a very high value (*C*_0_ to *C*_1_, with *C*_1_ ≫ *C*_0_) at *t* = 0. Figure 1(b) shows that when the sheet capacitance *C*_1_ increases, the eigenfrequency decreases, corresponding to a reduced phase velocity *ω*_1_/*β*. When *C*_1_ → +∞, the phase velocity approaches zero. More interestingly, the *group* velocity d*ω*/d*β* also tends to zero due to the extreme flatness of the dispersion curve. This method of wave freezing is very different from the conventional method for slowing down or freezing light by engineering the dispersion curve of photonic structures, where only zero group velocity is achieved while the phase velocity is in fact not reduced.

**Figure 3:**
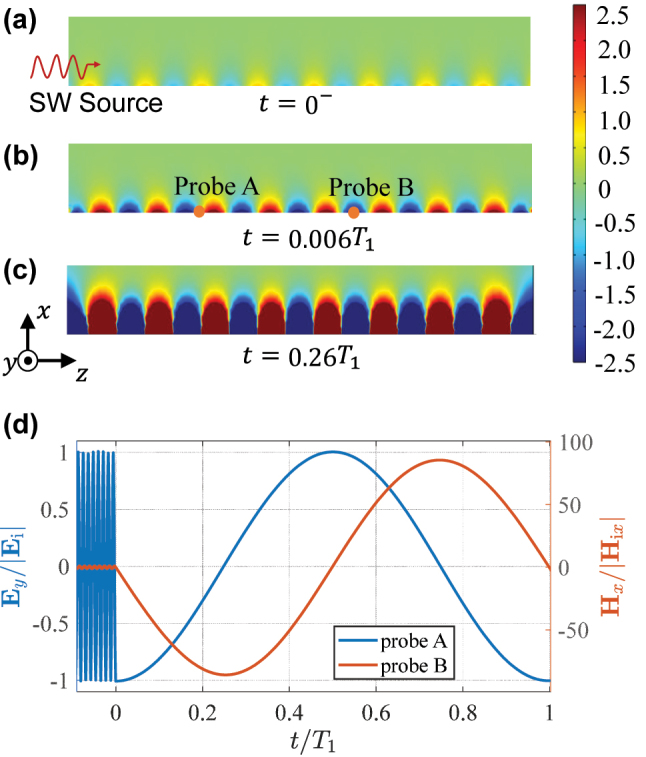
Spatial distributions of normalized magnetic fields **H**_*x*_/|**H**_i*x*_| at (a) *t* = 0^−^, (b) *t* = 0.006*T*_1_, and (c) *t* = 0.26*T*_1_. (d) Temporal variations of **E**_*y*_ at probe A and **H**_*x*_ at probe B.

In numerical examples, we assume *C*_0_ = 0.1 pF, *C*_1_ = 1000 pF, and *β* = 153 m^−1^. The corresponding eigenfrequencies are *ω*_0_/(2*π*) = 4.83 GHz and *ω*_1_/(2*π*) = 0.055 GHz. Figure 3 shows the distributions of **H**_*x*_ in the simulation space before and after the temporal jump. The magnetic field amplitude dramatically increases after the jump. This is the reason why the total energy of the system increases with a significant gain (*G* = 44). We position two probes [probe A and probe B in Figure 3(b)] on the boundary. Probe A is positioned at a maximum of **E**_*y*_ (also corresponds to a minimum of **H**_*x*_), and Probe B is placed at a maximum of **H**_*x*_. The time-varying electric field at probe A and magnetic field at probe B are recorded and shown in Figure 3(d). Noticeably, the oscillation frequency of fields after the jump (*t* > 0) is significantly reduced, while the propagation constant *β* remains unchanged, as seen in Figure 3(b). This result clearly demonstrates that the reflected and transmitted waves are ultra-slow. According to Eq. (6), the electric field amplitude of the reflected and transmitted waves can be estimated as *T*_E_ = 1/2 and *R*_E_ = −1/2. Thus, a standing wave is formed, with the amplitude |*T*_E_| + |*R*_E_| = 1 at the field maximum, which is confirmed in Figure 3(d). It is worth mentioning that waves cannot be completely stopped (reaching *ω*_1_ = 0) by this technique because in this case the energy becomes infinite, as predicted by Eq. (8).

### 4.3 Frozen surface waves

**Figure 4:**
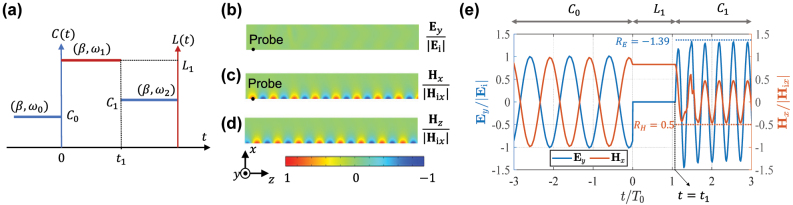
Frozen surface waves. (a) Boundary property as a function of time. Here, *C*_0_ = 1 pF, *L*_1_ = 0.1 nH, *C*_1_ = 0.1 pF, *β* = 153 (m^−1^), *ω*_0_ = 1.73 GHz, *ω*_1_ = 0 GHz, and *ω*_2_ = 4.83 GHz. (b)–(d) Spatial distributions of the static electric and magnetic fields at the freezing state. The field amplitudes are normalized by the incident-wave amplitudes. It is clear from the simulation that the magnetic field distributions of **H**_*x*_ and **H**_*z*_ have 90° phase difference, as predicted by the theory. (e) Temporal evolution of **E**_*y*_ and **H**_*x*_ at the probing point near the excitation side. The fields are normalized by their incident amplitudes.

Here, we investigate how surface waves behave when the boundary reactance changes from capacitive to inductive, and then it returns back to capacitive (*C*_0_ → *L*_1_ → *C*_1_), as shown in Figure 4(a). We will show that after the first jump, the surface wave completely stops, and the electrical fields disappear, while spatially non-uniform static magnetic fields remain frozen over the boundary. The electric field is re-created after the second jump, and the surface wave starts to propagate again.

Let us consider a capacitive boundary *C*_0_ over which a TE surface wave (*β*, *ω*_0_) propagates. The expressions for the incident electric and magnetic fields are found in Eq. (2). At *t* = 0, the boundary is suddenly changed to an inductive one, with the surface inductance *L*_1_, and the excitation is removed. Unequivocally, the existing TE surface mode cannot be supported by the inductive boundary which only supports TM surface modes. However, this statement holds only for an oscillating TE surface mode at a non-zero frequency. We notice that, if the frequency of the TE mode is converted to zero (*ω*_1_ = 0) after the jump, such static TE mode can satisfy the inductive boundary condition as well as the Maxwell’s equations in free space. Since the jump must conserve the *x*-component of magnetic field, we can write this component after the jump as $H_{x}^{t=0^{+}}=H_{0}{\text{e}}^{-\text{j}\beta z-\alpha_{1}x}x$ , where $H_{0}=-\frac{E_{0}\beta }{\omega_{0}\mu_{0}}$ . Note that the time-harmonic term ${\text{e}}^{\text{j}\omega_{1}t}$ disappears because *ω*_1_ = 0. Using Maxwell’s equations, the other field components can be determined as $H_{y}^{t=0^{+}}=0$ , $H_{z}^{t=0^{+}}=\text{j}H_{0}{\text{e}}^{-\text{j}\beta z-\alpha_{1}x}z$ , and $E_{x}^{t=0^{+}}=E_{y}^{t=0^{+}}=E_{z}^{t=0^{+}}=0$ . One can see that the electric field vanishes, and there is a static magnetic field which is spatially non-uniform both in *z* and *x* directions. Figure 4(b)–(d) shows the static field distribution in space when the boundary parameter is switched from *C*_0_ = 1 pF to *L*_1_ = 0.1 nH. Since the static magnetic field satisfies the boundary condition of the inductive boundary $\text{j}\omega_{1}L_{1}\cdot \left(n\times H_{z}^{t=0^{+}}\right)=E_{y}^{t=0^{+}}=0$ , it can be regarded as the “frozen eigenmode” of the inductive boundary. Figure 4(e) shows the instantaneous field variation at the probing point near the illuminating side (the probe position is shown in Figure 4(b) and (c)) for **E**_*y*_ and **H**_*x*_. One can see that the magnetic field stops to oscillate and remains constant in time after the boundary capacitance jumps to inductance, while the electric field quickly reduces to zero. Note that the electric field in Figure 4(e) is continuous in time. The sharp vanishing of electric field at *t* = 0 is caused by the ultra-short transient time. It is also important to mention that the inductance value affects the transient time. For small inductance like *L* = 0.1 nH used in this example, the transient time is very short and radiation to free space is negligible. If we increase the inductance, the transient time becomes longer and transient radiation is stronger. The theoretical analysis of the impact of inductance can use the Laplace transform method [22]. Here, we assume a small inductance value such that we can neglect the transient radiation.

At *t* = *t*_1_ = 1.076 *T*_0_, where *T*_0_ is the temporal period of the incident wave, the inductive boundary is switched back to a capacitive one with the surface capacitance *C*_1_ = 0.1 pF. The frequency of the surface mode must be converted to *ω*_2_ in order to satisfy the eigenvalue equation of the new capacitive boundary (*β*, *ω*_2_). Therefore, the vanished electric field revives after the second jump, generating reflection and transmission. By applying the temporal boundary condition of Eq. (5) at *t* = *t*_1_, i.e., $E_{ry}^{t=t_{1}^{+}}+E_{ty}^{t=t_{1}^{+}}=E_{y}^{t=t_{1}^{-}}=0$ and $H_{rx}^{t=t_{1}^{+}}+H_{tx}^{t=t_{1}^{+}}=H_{x}^{t=t_{1}^{-}}$ , we can calculate the reflection and transmission coefficients after the second jump:

(9) $$T_{E}=-R_{E}=\frac{\omega_{2}}{2\omega_{0}},\;T_{H}=R_{H}=\frac{1}{2},$$

where the coefficients are normalized by the incident amplitudes. The derivation details of Eq. (9) can be found in the Supplementary Information. The above equation indicates that the amplitude of the total electric field on the boundary can be amplified if *ω*_2_ > *ω*_0_, or equivalently *C*_1_ < *C*_0_. The normal component of magnetic field of the reflected and transmitted waves is always equal to half of the incident wave amplitude, independently of the value of *C*_1_. As shown in Figure 4(e), the amplitude of the reflected mode is very close to the theoretical predictions (marked as dashed lines).

From the duality theorem, we can also conclude that switching an inductive boundary to a capacitive one converts TM-polarized surface waves to static distributions of electric field, and this “frozen” static electric field can be converted again to a propagating TM surface wave by switching the surface reactance back to an inductive value.

It is worth mentioning that creation of a static magnetic field distribution at a temporal boundary was noticed in early works on wave propagation in a suddenly created plasma [5, 31, 32] and in magnetized plasma with quickly changed bias field [16, 33]. This mode was called *wiggler mode*. Here, in studying surface waves, we have found that a traveling wave can be completely converted into a static mode, which can survive independently. Furthermore, at a later time, it can be used for restoring the propagating wave when the second jump is made. This effect can be used to temporarily stop a light pulse.

### 4.4 Temporal metasurface prism

It is known that surface waves cannot be excited directly by free-space propagating waves due to mismatch of the propagation constants along the surface. The most conventional way to couple a propagating plane wave to surface waves relies on a prism where the tangential wavevector of the evanescent wave behind the fully reflecting interface can be brought to phase synchronization with the surface mode. In analogy, it is also possible to convert a free space wave to a surface wave via time-varying structures with uniform spatial properties [34–36]. In [34, 35], it is shown that a bulk plasma material with a sudden growth of plasma density can trap a plane wave and excite a surface wave. Here, we extend this concept to metasurfaces of infinitesimal thickness, and show that with an abrupt change of surface properties, the metasurface can transform free-space propagating waves to surface waves, acting as a temporal prism.

**Figure 5:**
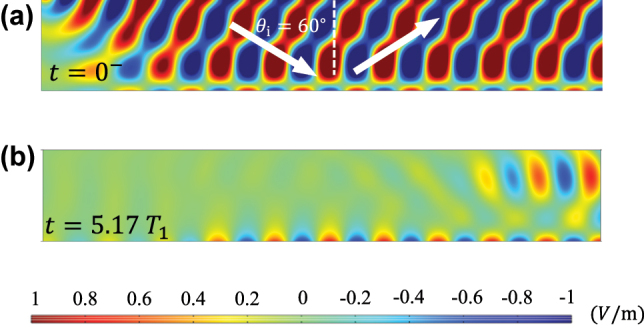
Temporal metasurface prism. (a) A standing plane-wave pattern (electric field) is formed above the boundary *C*_0_ before the temporal interface at *t* = 0^−^. (b) Plane waves are coupled to the surface wave after switching the capacitance to *C*_1_. The field is snapped at *t* = 5.17*T*_1_.

Let us consider a TE-polarized plane wave that is obliquely incident on a capacitive boundary *C*_0_ = 10 pF, as shown in Figure 5(a). The frequency of incidence is *ω*_0_/(2*π*) = 4.83 GHz, and the angle of incidence is *θ*_i_ = 60°. Since the incident tangential wavevector *β* = *k*_0_ sin *θ*_i_ (*k*_0_ is the free-space wavenumber) is not on the dispersion curve of the boundary, the incident wave does not couple to the surface mode and is fully reflected in the specular direction. As shown in Figure 5(a), above the boundary, there is an interference pattern of the incident and reflected plane waves. At *t* = 0, the boundary capacitance changes rapidly from *C*_0_ to *C*_1_ = 1 pF, and the excitation is removed. In order to adapt to the new boundary condition and excite a surface eigenmode of the boundary *C*_1_, the metasurface generates transition scattering. After the transient process, a surface mode (*β*, *ω*_1_) where *ω*_1_ < *ω*_0_ stays and propagates on the metasurface, since the boundary condition is satisfied, see Figure 5(b).

Depending on the capacitance value after the switch, the temporal jump creates fields at new frequencies that are positioned on the dispersion curve of the new boundary. Therefore, the velocity of the surface wave can be controlled by the capacitance of the new boundary. If the capacitance is very large, it can couple a propagating wave to ultra-slow surface waves.

## 5 Possible implementation strategies

**Figure 6:**
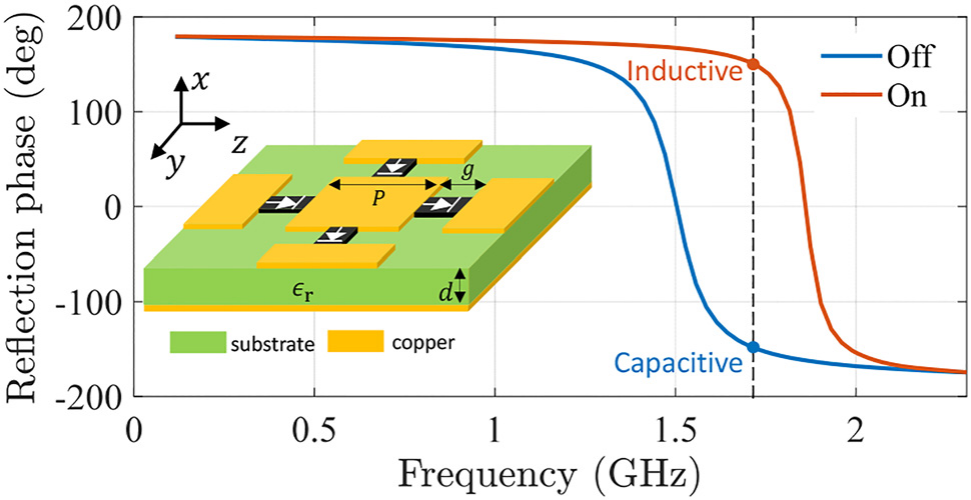
Reflection phases of a metasurface for diode states “Off” and “On”. The inset shows the metasurface structure with diode switches. The metasurface parameters are *ϵ*_*r*_ = 4.2, *P* = 50 mm, *g* = 5 mm, and *d* = 3.5 mm.

In actual implementations, time-varying metasurfaces are realized by dynamical control of meta-atom properties. Strictly speaking, a purely capacitive or inductive sheet cannot be realized since charge accumulation (modeled by capacitance) and current flow (modeled by inductance) always happen simultaneously in electromagnetic structures. In the microwave regime, a reactive metasurface can be implemented by using metallic patches positioned on a grounded substrate (see the insets in Figure 6). Such structures are characterized by an *LC* parallel resonant circuit [37]. Above the resonant frequency, the homogenized metasurface is capacitive. Integrating switchable elements, e.g., diode switches, between the adjacent meta-atoms can effectively modify the surface capacitance from one value to another [38–42]. For example, a narrow gap (between two metal patches) with inserted diode switches. If the diodes are reversely biased to the “off” state, the gap is nearly open-circuited, exhibiting high capacitance. If the diodes are in the “on” state, the gap is effectively shorted, and the surface capacitance is significantly reduced. In other words, the diodes can control the effective topology of metasurfaces, realizing sharp jumps of effective parameters. The transition time of diode switches should be much shorter than the oscillation period of the wave. Modern commercial microwave diodes, e.g., step recovery diodes, have transient time at picosecond level [38], allowing the metasurface to operate at tens of GHz.

Employing the same platform, it is also possible to switch the metasurface properties from capacitive to inductive. Figure 6 shows the reflection phases of an example of such metasurface. At the “off” state of the diodes, the resonant frequency (the reflection phase is equal to zero) is far below the surface wave frequency (marked as dashed straight line in Figure 6), and the metasurface exhibits a capacitive characteristic at the surface wave frequency. However, at the “on” state of the diodes, the resonant frequency jumps beyond the surface wave frequency and the metasurface shows an inductive behaviour. It is important to keep the surface wave frequency far away from the resonances of the structures at both “on” and “off” states, so that the metasurface can be approximated as a purely capacitive or inductive sheet. In this way, the effective surface impedance can be switched from capacitive to inductive and vice versa, realizing surface wave freezing and melting (the efficiency of this kind of realization should be carefully studied, but it is not within the scope of this paper). At optical frequencies, ultra-fast optical switching of material properties is possible (see, e.g., Ref. [43]). Therefore, by using this technique of switching of metasurfaces formed by subwavelength nanoparticles, the effective surface properties can be modified in a similar way as the microwave illustration.

## 6 Conclusions

To summarize, in this work we have studied surface waves supported by reactive metasurface boundaries that undergo fast changes of surface properties in time. It has been shown that such temporal jumps can efficiently control the speed and amplitude of scattered surface waves. Moreover, we have uncovered that a surface wave can be completely “frozen” by switching the surface reactance from capacitive to inductive, and, then, it is revived by jumping back to a capacitive boundary. In addition, we have also found that a temporal boundary can convert a free-space propagating wave to a surface wave. All these intriguing phenomena described in this paper (theoretically and numerically) explicitly indicate that temporal modulations of electromagnetic and optical systems give novel possibilities for controlling waves in the desired way. In the future, experimental validation of these phenomena is indeed an important research direction which needs to be explored. Finally, we would like to point out that the temporal discontinuous metasurface proposed in this work is fundamentally different from the programmable/reconfigurable devices (e.g., [44]) that do not rely on abrupt changes of surface parameters.

## Supplementary Material

Supplementary Material Details
