# Supplementary material for: Controlling surface waves with temporal discontinuities of metasurfaces
Source: Nanophotonics. 2023 Jan 24;12(14):2813–22. doi: 10.1515/nanoph-2022-0685 (PMC11501961; doi:10.1515/nanoph-2022-0685)
Supplement: Supplementary file 1 — Supplementary Material Details [file j_nanoph-2022-0685_suppl_001.pdf]

Supplementary material for the paper

"Controlling surface waves with temporal discontinuities of metasurfaces"

Xuchen Wang, Mohammad S. Mirmoosa, and Sergei A. Tretyakov

Contents

1 Derivation of Eq. (9) **2**

1 Derivation of Eq. (9)

When $t < 0$, there is only incident wave propagating on the capacitive surface, the incident electric and magnetic fields can be written as,

$$\mathbf{E}_\mathrm{i} = E_0 e^{-j\beta z - \alpha_0 x} e^{j\omega_0 t}\mathbf{y}, \quad \mathbf{H}_\mathrm{i} = \frac{E_0\alpha_0}{j\omega_0\mu_0} e^{-j\beta z - \alpha_0 x} e^{j\omega_0 t}\mathbf{z} - \frac{E_0\beta}{\omega_0\mu_0} e^{-j\beta z - \alpha_0 x} e^{j\omega_0 t}\mathbf{x}. \tag{S1}$$

At time $t = 0$, the surface switches to inductive. It is known that the inductive boundary cannot support a TE-polarized surface wave propagating on the surface. However, we notice that if the frequency is converted to zero, it satisfy the boundary condition, only if the electric field disappear. In this case, only the static magnetic field is allowed to survive on the surface. Suppose the x-component of magnetic field is conserved after the jump (after the transition scattering), we can write this component at $t = t_1^-$ as

$$\mathbf{H}_x^{t=t_1^-} = -\frac{E_0\beta}{\omega_0\mu_0} e^{-j\beta z - \alpha_1 x}\mathbf{x}, \tag{S2}$$

Note that the time-harmonic term $e^{j\omega_1 t}$ disappears because $\omega_1 = 0$. Using the Maxwell's equations in free space [S1, Chap. 10.2], the other field components can be determined as

$$\mathbf{H}_y^{t=t_1^-} = 0, \quad \mathbf{H}_z^{t=t_1^-} = -j\frac{E_0\alpha_0}{\omega_0\mu_0} e^{-j\beta z - \alpha_1 x}\mathbf{z} \tag{S3}$$

and

$$\mathbf{E}_x^{t=t_1^-} = \mathbf{E}_y^{t=t_1^-} = \mathbf{E}_z^{t=t_1^-} = 0. \tag{S4}$$

As one can see, the zero electric field and static magnetic field also satisfy the Maxwell's equation of free space.

After the second jump ($L_1 \to C_1$), the surface wave is melt and revived, generating reflected and transmitted waves. Assuming the reflection and transmission coefficients of electric field is R_E and T_E, respectively, with respect to the incident electric field, we can

write the electric and magnetic field after the second jump,

$$\mathbf{E}_{\mathrm{r}}^{t>t_1^+} = R_{\mathrm{E}} E_0 e^{-j\beta z - \alpha_2 x} e^{-j\omega_2 (t-t_1)} \mathbf{y}, \quad \mathbf{E}_{\mathrm{t}}^{t>t_1^+} = T_{\mathrm{E}} E_0 e^{-j\beta z - \alpha_2 x} e^{j\omega_2 (t-t_1)} \mathbf{y}, \tag{S5}$$

and

$$\mathbf{H}_{\mathrm{r}}^{t>t_1^+} = \frac{-R_{\mathrm{E}} E_0 \alpha_2}{j\omega_2 \mu_0} e^{-j\beta z - \alpha_2 x} e^{-j\omega_2 (t-t_1)} \mathbf{z} + \frac{R_{\mathrm{E}} E_0 \beta}{\omega_2 \mu_0} e^{-j\beta z - \alpha_2 x} e^{-j\omega_2 (t-t_1)} \mathbf{x}, \tag{S6a}$$

$$\mathbf{H}_{\mathrm{t}}^{t>t_1^+} = \frac{T_{\mathrm{E}} E_0 \alpha_2}{j\omega_2 \mu_0} e^{-j\beta z - \alpha_2 x} e^{j\omega_2 (t-t_1)} \mathbf{z} - \frac{T_{\mathrm{E}} E_0 \beta}{\omega_2 \mu_0} e^{-j\beta z - \alpha_2 x} e^{j\omega_2 (t-t_1)} \mathbf{x}. \tag{S6b}$$

The continuity of flux densities (Eq. (5) in the main text) at $t = t_1$ demands,

$$\mathbf{E}_{y}^{t=t_1^-} = \mathbf{E}_{\mathrm{r}y}^{t=t_1^+} + \mathbf{E}_{\mathrm{t}y}^{t=t_1^+} \quad \text{and} \quad \mathbf{H}_{x}^{t=t_1^-} = \mathbf{H}_{\mathrm{r}x}^{t=t_1^+} + \mathbf{H}_{\mathrm{t}x}^{t=t_1^+}. \tag{S7}$$

Substituting Eq. (S2), Eq. (S5), and Eq. (S6) into Eq. (S7), we obtain

$$R_{\mathrm{E}} = -\frac{\omega_2}{2\omega_0} \quad \text{and} \quad T_{\mathrm{E}} = \frac{\omega_2}{2\omega_0}. \tag{S8}$$

Knowing R_{E} and T_{E}, we can determine the reflection and transmission coefficients of x-component of magnetic field with respect to the x-component of incident magnetic field, i.e., $R_{\mathrm{H}} = T_{\mathrm{H}} = 1/2$.

References

[S1] D. K. Cheng *et al.*, *Field and wave electromagnetics.* Pearson Education India, 1989.
